# High Dose Vitamin E Attenuates Diabetic Nephropathy via Alleviation of Autophagic Stress

**DOI:** 10.3389/fphys.2018.01939

**Published:** 2019-01-21

**Authors:** Yuxue Zhao, Wenting Zhang, Qi Jia, Zhendong Feng, Jing Guo, Xueting Han, Yuning Liu, Hongcai Shang, Yaoxian Wang, Wei Jing Liu

**Affiliations:** ^1^Key Laboratory of Chinese Internal Medicine of Ministry of Education and Beijing, Dongzhimen Hospital Affiliated to Beijing University of Chinese Medicine, Beijing, China; ^2^Renal Research Institution of Beijing University of Chinese Medicine, Dongzhimen Hospital Affiliated to Beijing University of Chinese Medicine, Beijing, China

**Keywords:** diabetic nephropathy, autophagy, autophagic stress, vitamin E, high dose

## Abstract

It has been reported that autophagic stress, which is involved in many diseases, plays a key role in the development of diabetic nephropathy (DN). In this study, we investigated the effects of high dose vitamin E on renal tubular epithelial cells and autophagic stress-related mechanisms in diabetes condition. In diabetic rats, high dose vitamin E treatment significantly decreased the serum creatinine, urea nitrogen, urinary albumin and urinary protein, reduced the levels of LCN2, HAVCR1, LDH and 8-OHdG in urine, and attenuated the cellular apoptosis and interstitial fibrosis in renal cortex. *In vitro*, vitamin E could reduce the release of LCN2 and HAVCR1 and the protein levels of caspase 3 and TGF-β1, as well as improve the growth inhibition in cultured HK-2 cells after exposure to advanced glycation end products (AGEs). Also, LC3-II and SQSTM1-positive dots were significantly increased in the renal tubular epithelial cells of DN patients and diabetic rats, and in HK-2 cells after exposure to AGEs, which were markedly declined by vitamin E. In addition, we found that the autophagosome formation was not affected by AGEs, as assessed by the mRNA levels of LC3B, Beclin-1, and ATG7. However, AGEs blocked the lysosomal degradation of autophagosome, which was characterized by a decrease in the enzymatic activity of cathepsin B/cathepsin L and DQ-ovalbumin degradation in HK-2 cells, indicating that AGEs-induced accumulation of autophagic vacuoles was a sign of autophagic stress. Interestingly, vitamin E exerted a protective effect on lysosomes to reduce the autophagic stress. Taken together, we conclude that autophagic stress may play an important part in the progression of DN, and alleviation of autophagic stress though improvement of lysosomal function provides a promising novel approach for treating DN.

## Introduction

Diabetic nephropathy (DN) is one of the major microvascular complications of diabetes and a major cause of end stage kidney disease (Ahmad, 2015), imposing serious impact on morbidity, mortality and quality of life among the patients with diabetes. It causes glomerular damage, along with proteinuria, and subsequent tubulointerstitial lesions, leading to end-stage renal disease. Currently, therapeutic strategies for slowing-down DN progression are aimed at reducing hyperglycemia and intraglomerular pressure, and blocking RAS system. Unfortunately, many patients still experience progressive kidney function deterioration and inevitable loss of renal function.

Autophagy is a bulk degradation process that degrade damaged intracellular proteins and organelles. Accumulating evidences have shown that diabetic kidneys are deficient in autophagic activity, making kidney cells vulnerable to diabetes-associated damage. The accumulation of autophagic substrate p62/SQSTM1 has been observed in the kidneys of diabetic mice induced by STZ (Vallon et al., 2013), Wistar fatty rats (Kitada et al., 2011) and diabetic patients (Yamahara et al., 2013; Huang et al., 2014, 2016). Therefore, many researchers put forward views that autophagy has become a promising target for treating DN. As the study of autophagy develops in depth, the definition of autophagic stress gets researcher^,^ attention. Autophagic stress generally refers to a relatively sustained imbalance in which the rate of autophagosome formation exceeds the rate of its degradation (Chu, 2006). Studies have shown that the condition of autophagic stress is related to aging (Chu, 2006), hypoxia (Cimini et al., 2014) and many disease states, especially in response to protein or organelle damage (Brunk and Terman, 2002; Keller et al., 2004). We have reported that autophagic stress is involved in the mechanisms of podocyte damage in idiopathic membranous nephropathy (Liu W.J. et al., 2017). However, whether autophagic stress plays a role in DN has not been reported. And whether autophagic stress can be a new therapeutic target remains to be studied.

Vitamin E, a lipid-soluble vitamin with anti-oxidative and anti-inflammatory properties, is proposed for the prevention of kidney injuries associated with reactive oxygen species (ROS) (Liu P. et al., 2015), such as acute kidney disease induced by ischemic-reperfusion injury or exposure to nephrotoxic drugs (Dillioglugil et al., 2005; Tasanarong et al., 2013; Rezaei et al., 2016). Chronic inflammation and oxidative damage are important components involved in the pathogenesis of DN (Avci et al., 2014). Therefore, vitamin E supplementation is suitable for DN in theory. Indeed, the renoprotective effects of vitamin E by alleviating oxidative stress have been suggested in several experimental diabetic animals (Koya et al., 2003; Haidara et al., 2009; Hayashi et al., 2017). However, when the favorable effects of vitamin E treatment in animal models translated to diabetic humans, it becomes controversial. Therefore, further studies about the proper dose of vitamin E and the mechanisms underlying reno-protection are recommended to confirm its effects.

Autophagy and oxidative stress are intricately connected in kidney physiology and pathology. ROS can act as upstream regulators of autophagy induction. On the other hand, efficient autophagic activity can remove the damaged mitochondria and thereby indirectly prevent excessive ROS production and inflammasome activation and thus protect the kidney tissue. Previous researches have reported the effects of vitamin E and its derivative on autophagy, especially in tumor cells and neural cells (Cao et al., 2009; Li et al., 2012). So, the aim of our study was to determine: (1) the effects of high dose vitamin E treatment on DN; (2) whether the effects are related to alleviation of autophagic stress.

## Materials and Methods

### Patients

This study was approved by the Institutional Review Board of Dongzhimen Hospital Affiliated to Beijing University of Chinese Medicine. The clinical data from 17 patients (35–80 years) at the Affiliated Hospital of Guangdong Medical College and Dongzhimen Hospital Affiliated to Beijing University of Chinese Medicine were deidentified. Nine kidney tissue specimens in DN group were obtained from patients with biopsy-proven DN. Eight kidney tissue specimens in control group were obtained from patients with slight proteinuria or hematuria and characterized with a minimal change in histology.

### Animal Experiments

Male Sprague-Dawley rats (weighing 220–240 g) were accommodated in an animal house with temperature (22 ± 1°C) and lighting (12 h light–dark cycle) control. A single dose of 60 mg/kg streptozotocin (Sigma, St. Louis, MO, United States) was injected intraperitoneally to induce diabetes. Control rats were given injections of citrate buffer only. Rats with fasting blood glucose above 250 mg/dl were considered to be diabetes. Diabetic and non-diabetic rats were randomly divided into two groups (*n* = 6–7 per group) respectively, including control group (CON), vitamin E-treated group (Vit E), DM groups (DM) and vitamin E-treated DM group (DM + Vit E). Vitamin E (α-tocopherol; Sigma-Aldrich, Poole, United Kingdom) was given to the rats after the diabetic model was established successfully. Rats in the CON and DM groups were treated with 0.5 mL/kg corn oil (vehicle). Vit E and DM + Vit E groups were given 1000 mg/kg of vitamin E dissolve in corn oil via oral gavage. Body weight, food intake, and tail blood glucose were measured every 2 weeks after 8 h of fasting throughout the study period. At the 24^th^ week, HbA1c was measured by an aminophenyl-boronate-agarose affinity chromatographic method (Glyc-Affin GHb; Seikagaku Kogyo, Tokyo, Japan), and plasma insulin level was measured using an ELISA kit (Linco Research, Saint Charles, MO, United States). Experimental procedures were approved by the Ethics Committee of Beijing University of Chinese Medicine and performed in accordance with the National Academies Guiding Principles for the Care and Use of Laboratory Animals, 8th Edn.

### Urinary and Plasmatic Parameters

At the 12^th^ and 24^th^ week, blood samples were collected at 8 h post-meal via the tail vein and plasma was prepared. And the urine was collected for 24 h from each animal. Serum and urine levels of creatinine and urea nitrogen were measured by an automatic biochemistry analyzer (Olympus, Tokyo, Japan). Creatinine clearance was calculated by using an index of glomerular filtration rate. Urinary albumin concentration was assayed by a time-resolved fluorometric immunoassay (Feng Hua Bioengineering Corporation, China). Urinary HAVCR1, LCN2 (R&D Systems, Minneapolis, MN, United States), 8-hydroxy-deoxyguanosine (8-OhdG check; Shizuoka, Japan) levels were measured by ELISA kits. And urinary LDH level was assayed by using an automatic biochemical analyzer (Olympus).

### Kidney Cytoplasmic Lysate and Homogenate Analysis

At the 24^th^ week, all the experimental rats were sacrificed, renal cortices were rinsed and weighed, and the cytoplasmic fractions were prepared as previously described (Kuhad and Chopra, 2009). TGF-β1 was measured using the Quantikine Rat TGF-β1 immunoassay kit (R&D Systems, Minneapolis, MN, United States) according to the manufacturer’s instructions.

### Histological Analysis

Kidneys were fixed with 4% paraformaldehyde for 24 h and then embedded in paraffin. Two μm sections were stained with Masson’s trichrome to assess the fraction of the renal cortex occupied by interstitial tissue (INT%) as previously described (Liu Y.N. et al., 2017).

### Cell Culture and Treatments

Human proximal tubular HK-2 cells (ATCC, Manassas, VA, United States) were cultured in Dulbecco’s modified essential medium (DMEM/F12 at 1:1 radio) (Hyclone) supplemented with 10% fetal bovine serum (Gbico). The medium was routinely replaced every 2 days. Vitamin E (DL-α-tocopherol; Sigma-Aldrich, Poole, United Kingdom) was originally dissolved in 100% EtOH, and further diluted in growth medium to bring a final concentration of 0.1 mM. The cells were either treated with vitamin E for 24 h prior to 100 mg/ml non-glycated control bovine serum albumin (Co-BSA) or AGE-BSA (BioVision, Mountain View, CA, United States) addition. Then, the cell samples and culture supernatant were collected for the following experiment. The concentrations of LCN2 and HAVCR1 in culture supernatant were measured by Quantikine TM kits (R&D Systems). Quantification of TGF-β level was measured by ELISA kits (Invitrogen, San Diego, CA, United States). The activity of caspase-3 was determined by colorimetric caspase-3 assay kit (Abcam, Cambridge, MA, United States) in accordance with the manufacturer’s protocol.

### MTT Assay

The effect of vitamin E on the viability of HK-2 cells exposed to AGE-BSA was determined by MTT assay. Briefly, 96-well plates were plated in triplicate, with 2 × 104 cells per well. After 24 h of incubation, cells were treated with AGE-BSA and/or vitamin E for 24 h. Then 10 μL of MTT solution (5 mg/mL) was added to each well, and the cells were incubated for 4 h at 37°C. The medium was removed, 100 μL of isopropanol was added to each well, and the absorbance at 570 nm was measured.

### Enzymatic Assay

Fluorescence-based assay kits (BioVision, San Francisco Bay, CA, United States) were used to measure the activity of cathepsin B (CB), cathepsin D (CD), and cathepsin L (CL). After cleavage of the synthetic substrate by the cell lysate, the released fluorescence was quantified using a fluorescence plate reader according to the manufacturer’s instructions.

### Ovalbumin Dequenching Assay

HK-2 cells were incubated with 10 μg/mL DQ-ovalbumin (Invitrogen, Carlsbad, CA, United States) for an additional 2 h at 37°C. Then the cells were washed with PBS and fixed with 4% paraformaldehyde. The mean fluorescence intensity of green fluorescent DQ-ovalbumin puncta in individual podocytes was calculated and presented in the figures.

### Immunofluorescence and Immunohistochemical Study

Immunostaining examinations for tissues or cells was made as described previously (Liu et al., 2012). Rabbit anti-LC3B (Abcam, for cell), Rabbit anti-LC3B (Sigma, for tissue), SQSTM1/p62 (Santa Cruz), Alexa Fluor 594 goat anti-mouse IgG (Invitrogen), FITC-labeled goat anti-rabbit IgG (Santa Cruz), HRP labeled anti-rabbit mouse IgG antibodies (Zhongshan Goldenbridge Biotechnology) were used for tissue immunostaining assays. Immunofluorescence images were taken under TCS SP5 II confocal microscope (Leica Microsystems). The expression levels of LC3-II or SQSTM1 in 40 to 50 proximal renal tubule were first graded on a scale of 0 to 4, and the average of the scores was subsequently calculated.

### Real Time-Polymerase Chain Reaction

Total RNA was extracted by RNX-Plus solution (Takara Bio, Inc., Japan) according to the manufacturer’s instructions. The cDNA was synthesized at a final volume of 20 μL using cDNA synthesis kit (Takara Bio, Inc., Japan). Quantitative real-time polymerase chain reaction (qRT-PCR) analyses were performed using a SYBR Green mix in the Real-Time PCR System (Takara Bio, Inc., Japan). Relative gene expression data were calculated using the comparative threshold cycle method (ΔΔCt) with ACTB as housekeeping genes. To assess the specificity of each amplification, dissociation analysis was performed in every run. The primer sequences were as follows: LC3B forward, 5′-GGATATAGGTCACCCCTCAG-3′; LC3B reverse, 5′-GTTAAAGGAGTTCCTGTCACC-3′; Beclin-1 forward, 5′-CTGAAACTGGACACGAGCTTCAAG-3′; Beclin-1 reverse, 5′-TGTGGTAAGTAATGGAGCTGTGAGTT-3′; ATG7 forward, 5′-ATGCCAGGACACCCTGTGAACTTC-3′, ATG7 reverse, 5′-ACATCATTGCAGAAGTAGCAGCCA-3′.

### Western Blot Analysis

Western blot analysis was conducted as described previously (Liu et al., 2012). The primary antibodies against cleaved caspase-3 (Cell Signaling Technology, Beverly, MA, United States), α-SMA (Abcam), LC3B (Sigma), SQSTM1/p62 (Santa Cruz), tubulin (Abcam) and HRP-conjugated secondary antibodies (Beyotime Institute of Biotechnology, Jiangsu, China) were used.

### Statistical Analysis

All of the statistical tests were performed using SPSS 22.0. Data are presented as the means ± SEM. Two-group comparisons were performed using an independent sample *t*-test unless otherwise indicated. Multiple group comparisons were performed using analysis of variance followed by Bonferroni or Dunnett’s *post hoc* tests. Statistical significance was determined as *p* < 0.05.

## Results

### Vitamin E Ameliorated Proteinuria and Improved Renal Function of Diabetic Rats

The food intake, blood glucose and HbA1c levels were significantly increased, whereas the body weight and insulin levels were markedly decreased in diabetic rats compared with non-diabetic rats in week 24. However, these parameters were not significantly altered in vitamin E treated rats (Table 1). At weeks 12 and/or 24, serum creatinine, blood urea nitrogen, urinary albumin, urinary protein, albumin/creatinine, and kidney/body weight ratios were significantly increased, whereas creatinine clearance was significantly decreased in DM group compared to CON group. In diabetic rats, vitamin E treatment significantly decreased urinary albumin, urinary protein and albumin/creatinine ratios at week 12, creatinine and urea nitrogen were also significantly decreased at week 24 (Table 2).

**Table 1:** Effects of vitamin E on body weight, food intake, blood glucose, HbA1c and plasma insulin in non-diabetic and diabetic rats in week 24.

Group	CON	Vit E	DM	DM+Vit E
Body weight (g)	596 ± 28	588 ± 34	238 ± 16^###^	229 ± 13
Food intake (g/day)	26.7.4 ± 1.9	27.1 ± 2.1	49.5 ± 3.9^###^	48.1 ± 3.4
Blood glucose (mg/dl)	112 ± 4.62	107 ± 4.14	462 ± 3.65^###^	458 ± 7.14
HbA1c (%)	4.68 ± 0.19	4.35 ± 0.21	11.23 ± 0.87^###^	11.08 ± 0.91
Insulin (ng/dl)	1.99 ± 0.16	1.87 ± 0.19	0.34 ± 0.08^###^	0.39 ± 0.09

*Vitamin E treatment did not influence the body weight, food intake, blood glucose, HbA1c and plasma insulin in diabetic rats. ^###^p < 0.001 as compared with control (CON).*

**Table 2:** Effects of vitamin E on renal functions in non-diabetic and diabetic rats.

	Week	CON	Vit E	DM	DM+Vit E
Serum creatinine (mg/dl)	12	0.42 ± 0.07	0.49 ± 0.08	0.93 ± 0.10^##^	0.69 ± 0.09
	24	0.50 ± 0.07	0.54 ± 0.06	1.39 ± 0.19^###^	0.71 ± 0.10^**^
Blood urea nitrogen (mg/dl)	12	25.9 ± 3.2	25.2 ± 3.0	50.11 ± 6.5^##^	35.1 ± 4.4
	24	35.2 ± 3.9	33.5 ± 3.7	67.5 ± 6.9^###^	42.5 ± 4.1^**^
Urinary albumin (ug)	12	265 ± 28	251 ± 27	985 ± 112^###^	529 ± 60^***^
	24	289 ± 29	274 ± 28	1401 ± 153^###^	902 ± 88^**^
Urinary protein (mg)	12	11.7 ± 1.3	12.4 ± 1.4	22.4 ± 2.3^###^	13.8 ± 1.3^**^
	24	13.5 ± 1.6	13.0 ± 1.7	27.1 ± 2.8^###^	16.3 ± 1.7^**^
Albumin (ug)/creatinine (mg)	12	16.1 ± 1.7	17.2 ± 1.7	66.5 ± 6.8^###^	43.0 ± 4.8^**^
	24	21.1 ± 2.8	20.5 ± 2.4	88.1 ± 8.8^###^	55.0 ± 5.6^**^
Creatinine clearance (ml/min)	12	1.69 ± 0.15	1.66 ± 0.17	0.89 ± 0.10^##^	1.36 ± 0.15
	24	1.58 ± 0.16	1.66 ± 0.18	0.80 ± 0.10^##^	1.28 ± 0.15
Kidney/body weight^∗^1000	24	4.98 ± 0.56	4.85 ± 0.50	8.72 ± 0.88^##^	7.41 ± 0.76

*^##^p < 0.01 and ^###^p < 0.001 as compared with control (CON); ^∗∗^p < 0.01 and ^∗∗∗^p < 0.001 as compared with vehicle-treated diabetic control (DM).*

### Vitamin E Attenuated Renal Tubular Epithelial Cell (TEC) Injury in Diabetic Condition

**FIGURE 1:**
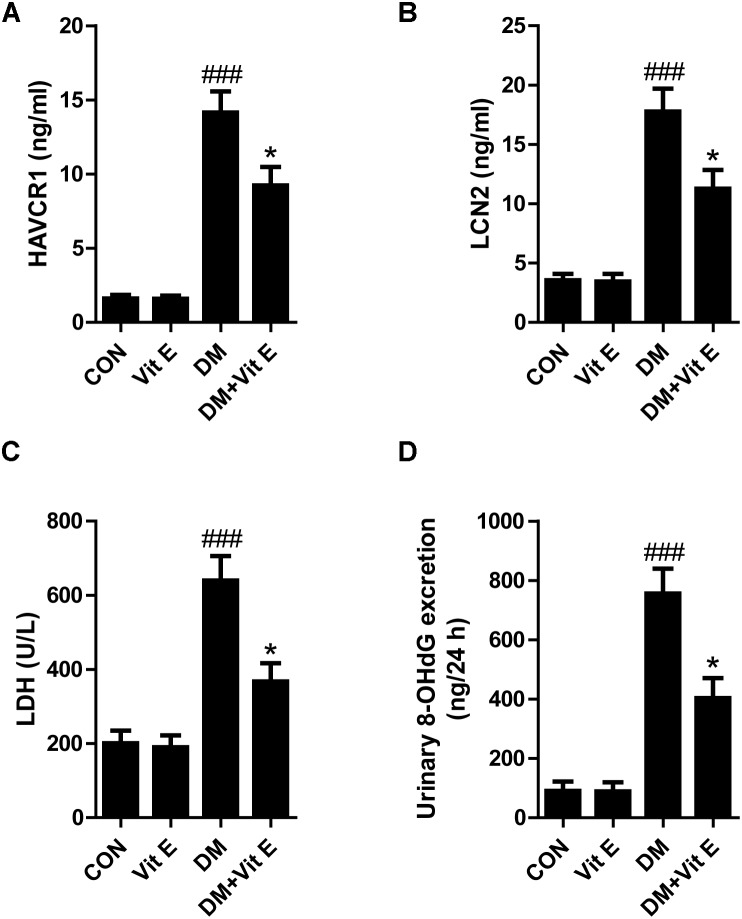
Effects of vitamin E on urinary HAVCR1, LCN2, LDH, and 8-OHdG excretions in non-diabetic and diabetic rats. **(A–D)** Bar graphs show the changes of HAVCR1, LCN2, LDH, and 8-OHdG excretions in urine in each group. ^###^*p* < 0.001 versus control (CON); ^∗^*p* < 0.05 versus vehicle-treated diabetic control (DM).

**FIGURE 2:**
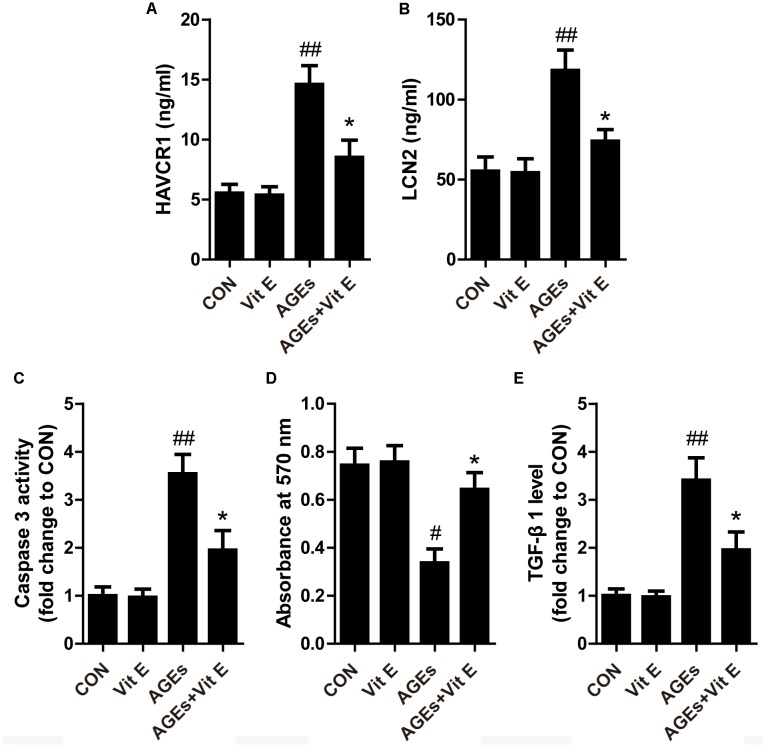
Effects of vitamin E on HK-2 cells injury induced by AGE-BSA. **(A,B)** The levels of supernatant HAVCR1, LCN2 were measured by ELISA. **(C)** The activity of caspase 3 was assessed by colorimetric caspase-3 assay kit. **(D)** The cell viability was assessed by MTT assay. **(E)** TGF-β1 level was measured by ELISA. ^#^*p* < 0.05, ^##^*p* < 0.01 versus Co-BSA (CON); ^∗^*p* < 0.05 versus AGE-BSA (AGEs).

Tubular injury is a critical component of the early course of DN (Vaidya et al., 2011; Bonventre, 2012). Tubular injury markers in the urine are early, sensitive, and specific markers of DN, even preceding the occurrence of microalbuminuria. Renal tubular injury markers HAVCR1, LCN2, LDH, and 8-OHdG were assessed by using ELISA in our study. As shown in Figures 1A–D, the urinary levels of HAVCR1, LCN2, LDH, and 8-OHdG were significantly higher in diabetic group than control group. However, vitamin E treatment significantly reduced the release of these markers. *In vitro*, AGE-BSA exposure enhanced the cellular HAVCR1 and LCN2 secretion, which was markedly reduced by vitamin E treatment (Figures 2A,B).

### Vitamin E Attenuated TEC Apoptosis and Interstitial Fibrosis in Diabetic Condition

**FIGURE 3:**
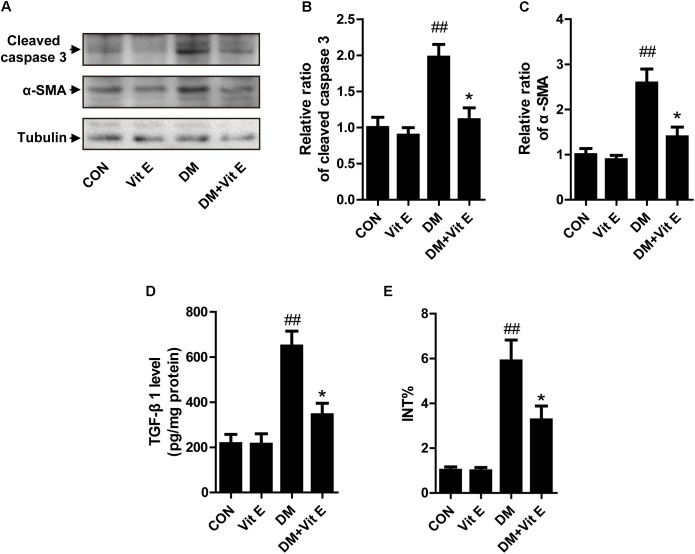
Effects of vitamin E on cellular apoptosis and interstitial fibrosis in the renal cortex of non-diabetic and diabetic rats. **(A)** The protein levels of cleaved caspase-3, α-SMA, and tubulin were analyzed by Western blot in the renal cortex of rats. **(B,C)** Densitometry was performed for quantification, and the ratio of cleaved caspase-3 or α-SMA to tubulin was expressed as fold of control. **(D)** TGF-β1 level was assayed in renal cortex lysates. **(E)** Bar graph shows the fraction of the renal cortex occupied by interstitial tissue (INT%). ^##^*p* < 0.01 versus control (CON); ^∗^*p* < 0.05 versus vehicle-treated diabetic control (DM).

Apoptosis of TECs is a major feature of DN. First, we utilized western blot to examine the expression of cleaved caspase-3 *in vivo*. As shown in Figures 3A,B, a significantly elevated cleaved caspase-3 protein level was noted in the renal cortex of DM group indicating increased cellular apoptosis. In cultured HK-2 cells, exposure to AGE-BSA also increased the cellular apoptosis and inhibited cell viability, characterized by an increased caspase-3 activity and reduced absorbance in the MTT assay, respectively (Figures 2C,D). Interestingly, vitamin E treatment significantly reduced the cellular apoptosis and improved cell viability *in vivo* and *vitro*. Interstitial fibrosis is essential element of DN, so we subsequently assessed the protein level of α-SMA and the fraction of the renal cortex occupied by interstitial tissue (INT%) in diabetic rats. The results showed that α-SMA protein level and INT% were higher in the diabetic rats than controls, which were markedly attenuated by vitamin E treatment (Figures 3A,C,E). TGF-β1, which had been reported to link epithelial–mesenchymal transition (EMT) and interstitial fibrosis, was also assessed *in vivo* and *vitro*. The similar pattern was observed (Figures 2E, 3D).

### Vitamin E Decreased the Accumulation of Autophagic Vacuoles and Autophagic Substrate in TECs in Diabetic Condition

**FIGURE 4:**
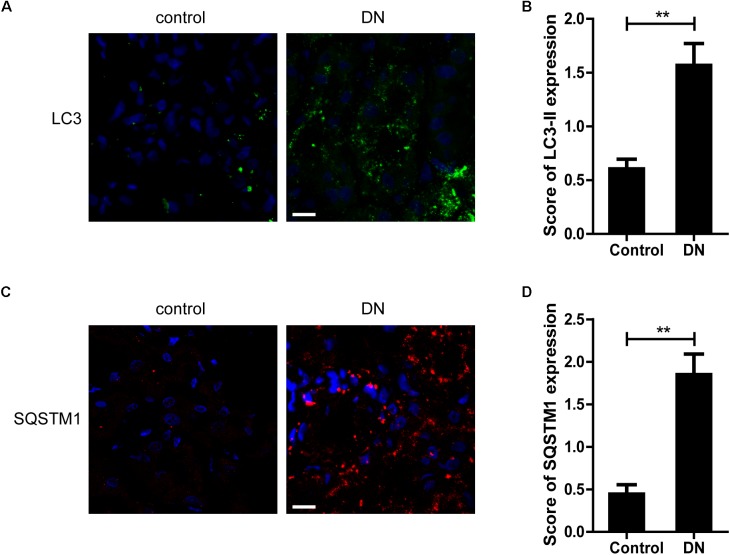
Quantitative changes in autophagic vacuoles and autophagy substrate in proximal tubules from DN patients or controls. **(A,B)** Immunofluorescent staining of LC3 and quantitative changes in autophagic vacuoles in renal tubules from DN patients or controls. **(C,D)** Immunofluorescent staining of SQSTM1 and quantitative changes in SQSTM1-positive puncta in renal tubules from DN patients or controls. Scale bar: 20 μm. ^∗∗^*p* < 0.01 versus control.

Next, we examined the expression of LC3-II, a key marker protein recruited into the autophagosome membranes during induction of autophagy, in TECs from patients with DN and controls. As shown in Figures 4A,B, more LC3-II-positive dots were observed in renal TECs from DN patients than controls. SQSTM1-positive aggregates, the specific target of autophagy degradation, was also increased in DN patients (Figures 4C,D). These results indicate that autophagic vacuoles are accumulated in TECs from DN patients.

**FIGURE 5:**
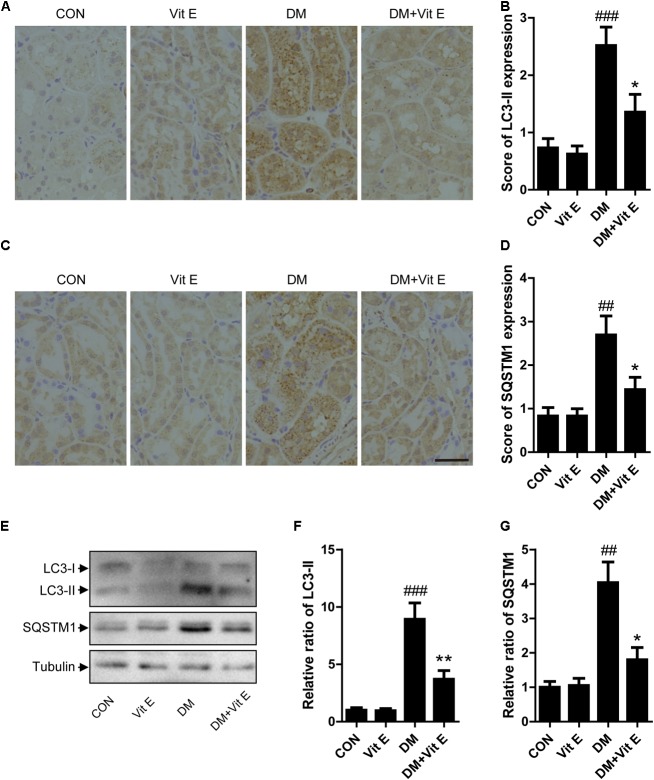
Effects of vitamin E on autophagic vacuoles and autophagy substrate in proximal tubules in non-diabetic and diabetic rats. **(A–D)** Immunohistochemical staining and expression score ofLC3-II and SQSTM1 in proximal tubules from non-diabetic and diabetic rats treated with vitamin E. **(E–G)** Western blot analysis of LC3, SQSTM1 level in the renal cortex from non-diabetic and diabetic rats treated with vitamin E. Densitometry was performed for quantification and the ratio of LC3-II, SQSTM1 to tubulin was expressed as fold of control. Scale bar: 40 μm. ^##^*p* < 0.05, ^###^*p* < 0.01 versus control (CON); ^∗^*p* < 0.05, ^∗∗^*p* < 0.01 versus vehicle-treated diabetic control (DM).

**FIGURE 6:**
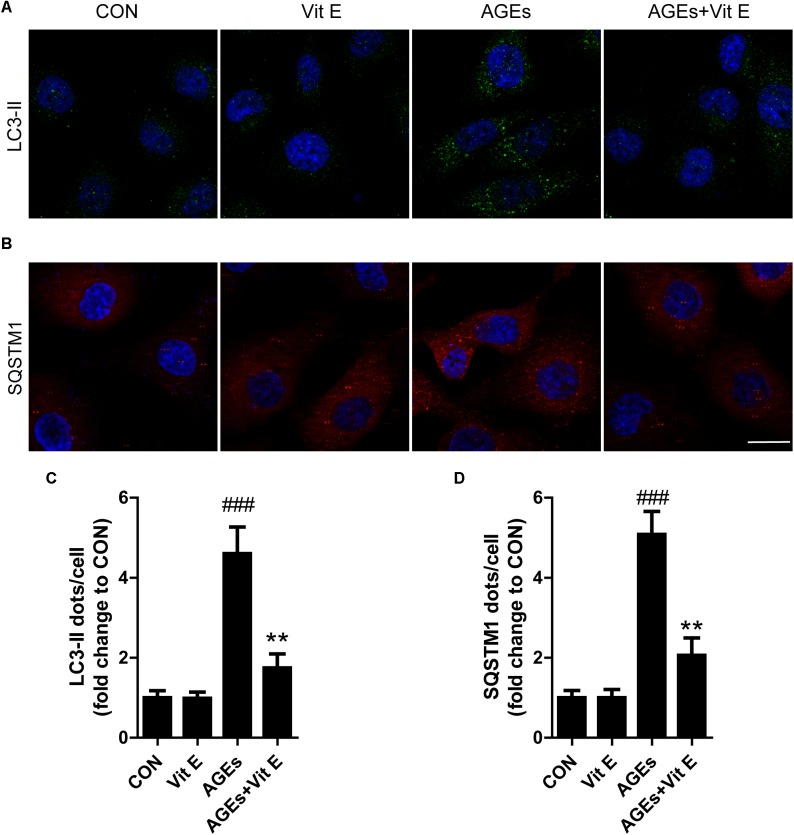
Effects of vitamin E on autophagic vacuoles and autophagy substrate in HK-2 cells after exposure to AGE-BSA. **(A,C)** Immunofluorescent staining of LC3 and quantitative changes in autophagic vacuoles in HK-2 cells after exposure toAGE-BSA. **(B,D)** Immunofluorescent staining of SQSTM1 and quantitative changes in SQSTM1-positive puncta in HK-2 cells after exposure to AGE-BSA. Scale bar: 20 μm. ^###^*p* < 0.001 versus Co-BSA (CON); ^∗∗^*p* < 0.01 versus AGE-BSA (AGEs).

To explore whether the renoprotective effect of vitamin E was associated with autophagy, the changes of autophagic vacuoles and autophagic substrate were studied both *in vivo* and *in vitro*. *In vivo*, immunohistochemistry analysis revealed that the amount of LC3-II and SQSTM1 puncta were significantly higher in diabetic rats than the controls (Figures 5A–D). Consistent with the immunohistochemistry, western blot analysis also revealed a significant increase in LC3-II and SQSTM1 protein levels in diabetic rats compared with the control rats (Figures 5E–G). Interestingly, vitamin E treatment attenuated the up-regulated expression of LC3-II and SQSTM1/p62. *In vitro*, immunofluorescent technology was performed to examine the expression of the LC3-II and SQSTM1/p62. As shown in Figure 6, exposed to AGE-BSA significantly increased LC3-II- and SQSTM1-positive puncta. Similarly, vitamin E treatment could reduce the puncta of LC3-II and SQSTM1/p62. These data suggest that the renoprotective effects of vitamin E may be achieved by reducing the accumulation of autophagic vacuoles and autophagic substrate.

### The Effect of Vitamin E on the Accumulation of Autophagic Vacuoles Might Be Attributed to a Decrease in Autophagic Stress

**FIGURE 7:**
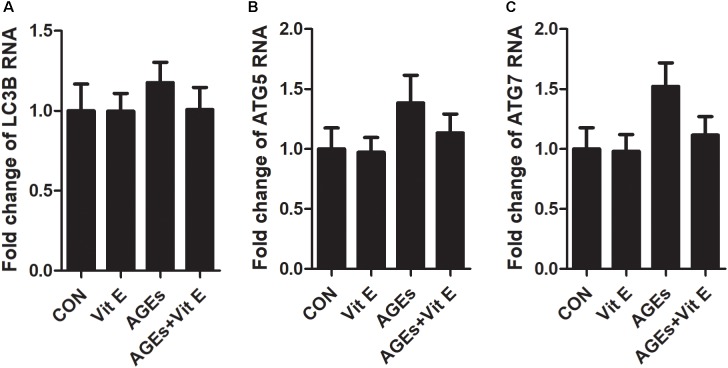
Effects of vitamin E on mRNA levels of LC3B, Beclin-1, and ATG7 in HK-2 cells after exposure to AGE-BSA. The relative mRNA expressions of LC3B **(A)**, Beclin-1 **(B)**, and ATG7 **(C)** after exposure to AGE-BSA. There was no significantly difference observed.

**FIGURE 8:**
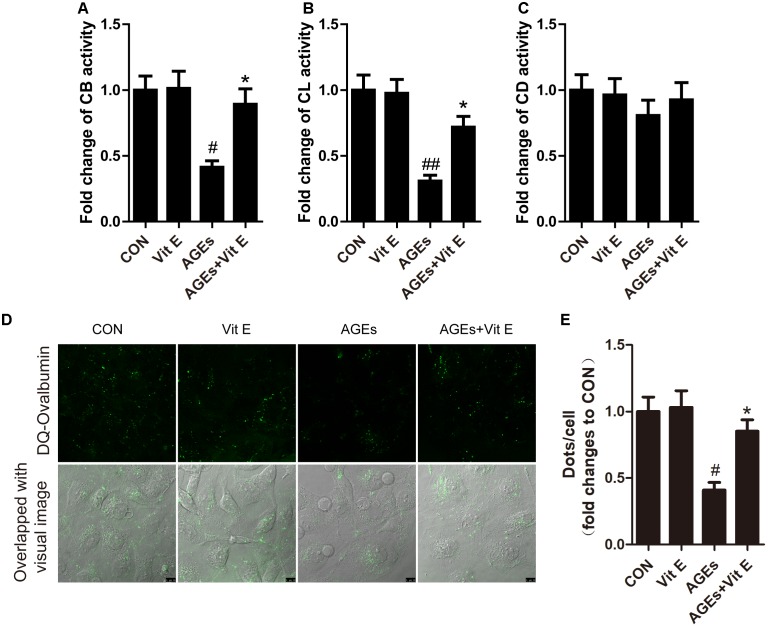
Effects of vitamin E on enzymatic activity and lysosomal degradation of DQ-ovalbumin in HK-2 cells after exposure to AGE-BSA. Proteolytic activity of cathepsin B **(A)**, cathepsin L **(B)**, and cathepsin D **(C)** in HK-2 cells exposed to AGE-BSA. **(D,E)** Cleaved fluorescent DQ-ovalbumin (green) and relative DQ-ovalbumin vesicles in HK-2 cells exposed to AGE-BSA. Scale bar: 10 μm. ^#^*p* < 0.05, ^##^*p* < 0.01 versus Co-BSA (CON), ^∗^*p* < 0.05 versus AGE-BSA (AGEs).

**FIGURE 9:**
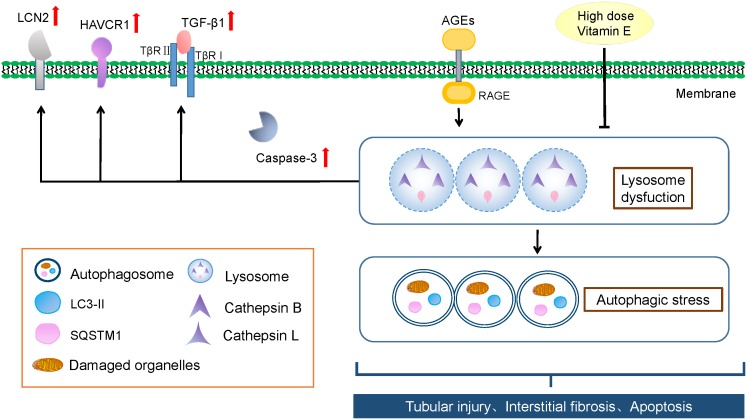
Schematic representation of vitamin E’s renoprotective effect and the autophagic stress-associated mechanisms. AGEs induces autophagic stress by blocking lysosomal-dependent degradation of autophagosomes in proximal tubules in DN, irrespective of autophagosome formation. And the accumulation of autophagic vacuoles and autophagy substrate is likely a sign of increased autophagic stress. High dose vitamin E could attenuate TEC injury and prevent the progression of DN by depressing autophagic stress.

Autophagic stress, defined as a relatively sustained imbalance between autophagosome formation and degradation, plays a key role in autophagy-related diseases. To explore whether the accumulation of autophagic vacuoles was a sign of autophagic activation or a hint of autophagic stress, the upstream molecules of the autophagy pathway were subsequently studied by examining the mRNA expression of LC3B, Beclin-1, and ATG7 *in vitro*. We found that the mRNA level of LC3B, Beclin-1, and ATG7 were not significantly increased after exposure to AGE-BSA, and vitamin E treatment had no effect on the mRNA expression (Figure 7), suggesting non-effect on autophagic induction and autophagosome formation. The lysosomal-mediated degradation systems are essential for the degradation of autophagosome. Therefore, we next examined the activity of lysosomal proteolytic enzyme. Compared with control group, there was a significant decrease in CB and CL activity after exposure to AGE-BSA (Figures 8A,B), although CD activity was not changed significantly (Figure 8C). DQ-ovalbumin, a self-quenched substrate for proteases, was subsequently used to evaluate the digestive function of lysosomes. As shown in Figures 8D,E, there was a significant drop in the DQ-ovalbumin dots per cell after AGE-BSA exposure as assessed by fluorescence. However, the enzymatic activity of CB/CL and DQ-ovalbumin vesicles in AGE-treated HK-2 cells were increased after vitamin E treatment, suggesting an improvement of lysosomal function (Figures 8A–E). These results indicate that the accumulation of autophagic vacuoles is attributed to an increase in autophagic stress, but not autophagic activation. And the renoprotection of vitamin E is closely associated with the reduction of autophagic stress by increasing autophagosome degradation (Figure 9).

## Discussion

In the present study, we investigated the effects and autophagic stress-related mechanisms of high dose vitamin E in TECs in diabetic condition. Although vitamin E treatment did not improve glycemic control, it offered hypoalbuminuric, hypoproteinuric, urea nitrogen-decreasing, and renoprotective effects. In addition, vitamin E treatment could alleviate TEC injury, apoptosis and prevent progression of EMT and tubulointerstitial fibrosis. To mimic diabetes-associated damage in TECs, human proximal tubular cell line HK-2 was treated with AGEs, because protein glycosylation for AGEs in hyperglycemia is a major cause of diabetic complications. And similar results were observed *in vitro*.

Since oxidative stress and inflammation have been demonstrated a significant role in development and progression of DN, quite a few studies indicate favorable results of vitamin E on albuminuria or renal function in DKD. For example, high doses (1,800 IU/day) of vitamin E treatment appeared to be effective in improving renal function in patients with type 1 diabetes for less than 10-year duration (Bursell et al., 1999). Oral high-dose (1,200 IU/day) vitamin E for 12 weeks among DN patients had favorable effects on biomarkers of kidney injury, inflammation, and oxidative stress (Khatami et al., 2016). Vitamin E (680 IU) combined with vitamin C (1250 mg) per day, reduced albumin excretion rate by 19% in Type 2 diabetic patients with persistent micro/macroalbuminuria (Gæde et al., 2001). Tocotrienols, isoforms of vitamin E, ameliorated proteinuria and protected the kidney against inflammation and nitrosative stress in T2DM patients (Haghighat et al., 2014). However, these studies are of relatively short duration and small sample size. In contrast, in the Heart Outcomes Prevention Evaluation (HOPE) trial, which included more than 3,600 people with diabetes, some of whom already displayed microalbuminuria, treatment with 400 IU vitamin E per day for an average of 4.5 years had no effect on cardiovascular outcomes or nephropathy (Lonn et al., 2002). One of the striking differences between HOPE trial and other clinical studies is the doses of vitamin E. Previous study has shown a significant linear trend between dosage of vitamin E and percent change of F2-isoprostanes, a biomarker of free radical mediated lipid peroxidation (Roberts et al., 2007). Thus, administration of 400 IU vitamin E in the HOPE trial may be too low to have an effect on DN. So, in our study, we gave a high dose of vitamin E (1000 mg/kg) to the rodent models.

It is mainly associated with the alleviation of oxidative stress about the mechanism underlying vitamin E’s protection for DN in earlier studies (Koya et al., 2003; Haidara et al., 2009; Kuhad and Chopra, 2009). Whether attenuation of autophagic stress is involved has not been elucidated. Autophagy activity is relative low in proximal tubular cells, but a basal level of autophagy is required to sustain TECs homeostasis under physiological conditions. Some studies indicate that impairment of autophagy in TECs is implicated in the pathogenesis of DN (Shinji and Daisuke, 2015). Previous studies, including our own, have shown that autophagic vacuoles are increased in TECs during the progression of DN (Huang et al., 2014, 2016; Liu W.J. et al., 2015). It may be resulted by autophagic induction, which promotes autophagosome formation. Also, it can be attributed to a blockage of autophagosome degradation. In the present study, we found the balance between the autophagosome formation and degradation was disrupted by the dysfunction of lysosomal-dependent degradation pathway, suggesting that the accumulation of autophagic vacuoles is a sign of autophagic stress. Interestingly, vitamin E could decrease the autophagic stress by activating autophagy after exposure of AGEs to HK-2 cells, since not only autophagic vacuoles but also autophagic substrate was down-regulated. More importantly, vitamin E could increase the enzymatic activities and the lysosomal degradation, indicating a recovery of lysosomal-dependent autophagosome degradation. Different from CB and CL, CD could remain active at neutral pH after leakage into cytoplasm (Boya and Kroemer, 2008), which might explain the unchanged CD activity in our study. As an antioxidant, does vitamin E reduce autophagic stress through its anti-oxidation effect? It is well-known that autophagy and oxidative stress are intricately connected. Generally, ROS act as the autophagy inducer in the upstream of autophagic pathway (Scherz-Shouval and Elazar, 2011). However, AGEs and vitamin E did not influence autophagic induction significantly, as assessed by LC3B, Beclin-1, and ATG7 mRNA expression in our study, suggesting that the anti-oxidative effect of vitamin E does not alleviate autophagic stress by reducing the formation of autophagosomes. The study about senescence revealed that oxidative stress-induced autophagy impairment was closely associated with the reduced degradation capability of lysosomes (Tai et al., 2017). Our previous study also supported that AGEs-RAGE axis-evoked oxidative stress played an important role in a lysosomal impairment, and antioxidant could improve the lysosomal dysfunction (Liu W.J. et al., 2015), which indicate that antioxidant may reduce autophagic stress though increasing the degradation of autophagosomes. Therefore, we speculate that the suppressive effect of vitamin E on autophagic stress may be closely associated with its attenuation of oxidative stress.

Taken together, we showed that TECs was undergoing autophagic stress during the progression of DN. In addition, we revealed that alleviation of autophagic stress was included in vitamin E-induced amelioration of DN. Autophagic stress might be an important therapeutic target for DN.

## Author Contributions

WL, YW, and YL designed the experiments. YZ, WZ, QJ, ZF, JG, and XH carried out the experiments. HS and YZ analyzed the experimental results. YZ and WZ revised the manuscript.

## Conflict of Interest Statement

The authors declare that the research was conducted in the absence of any commercial or financial relationships that could be construed as a potential conflict of interest.
